# Parents’ expectations and experiences of the 6-week baby check: a qualitative study in primary care

**DOI:** 10.3399/bjgpopen20X101110

**Published:** 2020-11-18

**Authors:** Gill Gilworth, Sarah Milton, Angel Chater, Irwin Nazareth, Andreas Roposch, Judith Green

**Affiliations:** 1 Department of Population Health Sciences, King's College London, London, UK; 2 Centre for Health, Wellbeing and Behaviour Change, University of Bedfordshire, Bedford, UK; 3 Department of Primary Care & Population Health, University College London, London, UK; 4 Great Ormond Street Institute of Child Health, University College London, London, UK

**Keywords:** general practice, parents, qualitative research, infant

## Abstract

**Background:**

The Newborn and Infant Physical Examination (NIPE) programme requires all babies to have a comprehensive health check at 6–8 weeks of age. These are typically completed by GPs. Although person-centred care has achieved prominence in maternity care policy in recent years, there is limited empirical evidence on what parents and/or carers expect from the check, and how far experiences meet their needs.

**Aim:**

To explore the expectations and experiences of parents attending their GP for a baby check.

**Design & setting:**

A qualitative study was undertaken in primary care in London.

**Method:**

Content analysis was undertaken of transcripts of semi-structured interviews. Interviews were conducted with a total of 16 participants (14 mothers and two fathers) who had recently attended for a 6-week check for their baby.

**Results:**

Despite the availability of plentiful sources of general advice on infants’ health and development, a thorough check by a trusted GP was an important milestone for most parents. They had few specific expectations of the check in terms of what examinations were undertaken, but even experienced parents anticipated reassurance about their baby’s normal development. Many also hoped for reassurance about their own parenting. Parents appreciated GPs who explained what they were doing during the examination; space to raise any concerns; and combined mother and baby checks. Referrals to secondary care were generally experienced as reassuring rather than a source of anxiety.

**Conclusion:**

The baby check meets needs beyond those of the NIPE screening programme. Protecting the time for a thorough consultation is important for parents at what can be a vulnerable time.

## How this fits in

The primary purpose of the 6-week baby check in primary care is screening for common abnormalities. Even for experienced parents, it is also an important milestone, meeting a range of other needs. The key ones are reassurance about their infant’s normal development, and reassurance that they are doing well as parents. GPs are also well placed to recognise emotional concerns of new parents. Despite time pressures for GPs, parents appreciate GPs who explain examinations as they do them, and who provide time for raising additional concerns about both their baby’s and their own health.

## Introduction

The NIPE programme was established in 2008 to improve the quality, timeliness, and consistency of examinations for newborns and infants. The four principal screening components are the eyes, heart, hips, and testes for males. The guidelines were revised in August 2019.^1^ They specify all eligible babies will be offered an examination within 72 hours of birth and at 6–8 weeks of age (the 6-week check), given that some abnormalities not easily detected in newborns can become more apparent by this age.^2,3^ The 6-week check is done in primary care, typically by GPs. Guidance advises GPs to include the baby’s height, weight, head circumference, general tone and posture, lungs, and general wellbeing, and to provide a range of general health promotion advice, covering topics as diverse as car safety, breastfeeding, and smoking.^4^ The National Institute for Health and Care Excellence (NICE) also advises that at each postnatal contact healthcare professionals should *'* *offer consistent information and clear explanations to empower the woman to take care of her own health and that of her baby, and to recognise symptoms that may require discussion* *'*.^5^ Also, in line with broader policy encouraging person-centred care, *'* *a* *person-centred, integrated approach to providing services is fundamental to delivering high-quality care to women, babies, partners* *,* *and other family members* *'*.^5^

To achieve its many aims, the 6-week check is often at least twice the length of a usual consultation. In the context of increasing demands on time in primary care,^6–8^ and widely reported pressures on the primary care workforce,^9^ the time devoted to this check may be coming under threat, given debates around the need for ‘standard’ consultation lengths.^6^ Debates about extending NIPE training to other members of the primary care workforce attest to this pressure.^10^ The 6-week check has also come under some scrutiny for its screening effectiveness.^11^ There is little evidence on parents’ views of the 6-week check, and whether it meets their needs. One study of postnatal care in general reported parents’ positive views of the 6-week check.^12^ The Care Quality Commission maternity services surveys focus primarily on choice in antenatal care, the labour, and birth: recent surveys have found women report poorer experience of care postnatally, compared with antenatal care and care during labour and birth.^13^

Given the policy imperatives for clinicians to better understand service provision from the perspective of patients (in this case, parents and/or carers), limited evidence on parents’ views, and the context of increasing pressure on primary care workloads, it is timely to ask whether the 6-week check does meet the needs of parents, and whether it justifies a long appointment with a GP. Therefore, a qualitative study of parents’ expectations and experiences was undertaken. This article covers an important topic from the parents’ perspective that has not been well researched previously.

## Method

### Setting and participants

Participants were recruited from eight GP practices in north London providing health care for people from diverse socioeconomic backgrounds. The practices have different arrangements for the checks: six combine the mother’s postnatal check-up with the 6-week check (in a 30-minute appointment); one schedules checks at 8 weeks so they can be followed by a vaccination appointment; and one allocates 30 minutes exclusively for the baby check, completed jointly by a nurse and GP.

A researcher provided parents waiting for their 6-week check with written study information. Those expressing interest were followed-up by phone to provide an opportunity for questions. Inclusion criteria were being aged >16 years, with sufficient fluency in English to read and understand the written information, and participate in an interview conducted in English. The 16 participants were 14 mothers and two fathers, aged 23–41 years. Ten described their ethnicity as White British, four as non-British European, and two as Black or Minority Ethnic background. Participants’ parenthood experiences ranged from six first-time parents, to two attending with their sixth child.

### Data collection and analysis

Fourteen semi-structured interviews were carried out between 2018 and 2019. This was a convenience sample from parents attending for baby checks at GP practices participating in a project relating to the hip part of the NIPE examinations, which will be reported separately. Twelve interviews were with the mother only, two included the baby’s mother and father. Parents gave written consent prior to the interview. A £25 shopping voucher was offered in acknowledgement for the time to take part in an interview. An interview topic guide prompted for: experience of healthcare services since the baby’s birth; expectations and prior knowledge of the check; the process of arranging the appointment; and experiences of the 6-week check. Interviews lasted between 20 and 54 minutes (mean 39 minutes). They were conducted by GG or SM, both female post-doctoral qualitative researchers. They took place in the parents’ homes (*n* = 12) or by phone (*n* = 2), 1–5 weeks after the check. Interviews were audio-recorded and transcribed for analysis. Thematic content analysis used a mix of deductive and more inductive approaches,^14^ with an initial mapping of recurrent themes around expectations and experiences of the 6-week check (including the following topics: parents’ reported needs from the check-up; their expectations; and experiences of communication with GPs and other members of practice staff). Initial codes were generated by the first author and revised following discussion between GG and SM, who had conducted the interviews, and JG who was also involved in data analysis. The themed data extracts were then analysed in detail, using line-by-line coding, comparisons, and further discussions between the team. Data organisation was assisted by the use of NVivo software (version 12).

## Results

This article reports three key themes in parents’ accounts: their reported expectations of the 6-week check; the importance of the check in the light of available sources of knowledge about infant development and health; and their reported positive and negative experiences of the consultation.

### Parents’ expectations of the 6-week check

The first weeks of an infant’s life are exhausting times for most parents. Participants noted that the 6-week check comes at a point when they may be struggling with sleep, emotions, concerns about the baby, and managing everyday life, whether they are first-time parents or more experienced. As one participant (PA2, a mother of four), put it: *'I think everyone’s drowning at 6* *weeks.'* Parents ranged from those whose expectations were for a routine, unremarkable appointment, to be fitted into a busy schedule, to those who had more concrete expectations of an opportunity to discuss concerns about their own or their infant's health. Few knew in detail what to expect in terms of the specific examinations undertaken. None described the check in terms of its screening function: it was typically understood as a ‘general’ check of the baby’s health and development:

*'I did my research online checking what’s going to happen on that day so I can kind of prepare myself for her.'* (PA4, first-time father)

*'Obviously I understand it’s a routine check-up, fine, but you're not explained what that entails … there probably was a pamphlet somewhere saying what it did entail, but you get so much information, don't you, when you give birth … maybe I missed it ... you just want everything to be OK with your baby so it’s a bit, you want it to be done with, and hopefully everything’s OK.'* (PA8, mother of 2)

*'I don't think I really had expectations, um, just, I guess just to be updated on how she is and everything’s normal ... maybe that’s part of being a busy mum, you just have an appointment, you go to it.'* (PA11, mother of 4)

Only one mother expressed an explicit desire for more information prior to the appointment about what to expect:

*'... if I’d have thought about it and I knew ... what they were looking for and what was being examined, I might have actually thought about other things probably. It’s much harder to think on the spot isn’t it? ... So I definitely think more information beforehand — that’s probably the thing that could make it a bit better from my perspective.'* (PA3, first-time mother)

Others had checked first if they were interested, or suggested that they had no need to know specifically what the GP was looking for:

*'No, I haven’t seen it* [what the GP checks for], *I don’t need to see it. Six weeks must mean they need to check something. If I go on the NHS website they probably would have a description.'* (PA5, mother of 2)

However, despite having few detailed expectations that could be articulated about the tests or examinations that were to be done, most parents did have clear expectations about one outcome of the consultation: that it would provide reassurance. A common theme was that of the check as a marker or threshold in an infant’s life. It was the first time, for some, that the baby would be ‘presented’ to those outside the immediate family. This meant that even for experienced parents, the appointment could be more significant than simply a check-up. For instance, PA2 (a mother of four), noted that *'from my last experience I knew what was to be expected*', but, like others, she still described the appointment as *'a bit daunting … I’m handing him over to let somebody else have a good old check at him and scrutinise him*'.

The expectation of, and desire for, reassurance was common to almost all participants. First, most reported wanting reassurance that their baby is healthy and developing normally:

*'I do think it’s good they have it because it is reassuring, just so you know you’re along the right lines, he’s doing OK.'* (PA3, first-time mother)

*'I think it put our mind at ease,* [pause] *I felt very comfortable, like, happy, more than happy to have her checked, yeah, so I was really happy with the check and with presenting* [baby’s name]*.'* (PA4, first-time mother)

Second, even the more experienced parents also appreciated reassurance that they were doing well, as parents:

*'... one of my other children … it was just, like, much quicker and I thought afterwards I wish I could have said to the GP, "You know, some mums are coming in here this is the only interaction they're going to have, tell them they're doing an amazing job" ... I can remember when she first gained weight … and they were like "Oh mum, you must be doing a great job," and I was like, "You know, I’m a six-time mum, I know I'm doing a fine job," but it’s still nice to hear it from someone else.'* (PA12, mother of 6)

*'I was also a lot more anxious at that stage so, yeah, it would have been really nice to come away, she didn’t even say,"You're doing a good job" or anything.'* (PA13, mother of 2)

However, this reassurance was less useful if offered merely as a minimisation of expressed parental concerns. This mother reported that her GP’s response had ignored her needs, which were for more than reassurance:

*'I wanted to tell the GP also that I was experiencing anxiety ...* [pause] *the GP was a man and he just told me that both the* [baby’s] *congestion and the nappy rash and the anxiety were completely normal and nothing to worry about ... he was very dismissive.'* (PA13, mother of 2)

### The check in the context of information sources

Parents reported accessing a large range of information sources about infant development and health problems. These included the internet, friends and family, and books and leaflets provided by the NHS. Although many reported a routine scepticism about internet sources of advice (*'I would rely on people more than the internet always'* [PA12, mother of six]; *'it fills your head with so much stuff, you kind of lose any sense of intuition or anything, it’s much better to actually talk to people'* [PA13, mother of two]), several internet sources were, in practice, widely drawn on, including NHS websites, general Google searches, and named community and commercial sites such as Mumset, WonderWeeks and Bounty:

*'They send you an email each week, like, you know your baby at, like, 6 weeks, 7 weeks, 8 weeks, 9 weeks … what to expect a baby to be doing at that point … it’s very useful.'* (PA12, mother of 6)

*'I've been getting a lot of information for that, which makes anxiety a lot worse, because then you’ve read every single possibility. Like, every side effect of Infant Gaviscon.'* (PA13, mother of 2)

Although there were comments about inducing anxiety by searching online, many clearly regularly turned to internet searches for information and reassurance on minor problems and developmental milestones:

*'I just find it quicker to just get my answers from Google and, yeah, NHS website ... the slightest concern I straightaway look up and, you know, once you hear other mothers, other kids have had it you just feel assured, um, let’s say at the time when she wasn’t smiling, all my others were quite, like, quite close to the milestone when they're supposed to, so, um, straightaway* [I] *looked that up.'* (PA11, mother of 4)

Given the range of available resources for parents, and the need identified above for reassurance, the challenge is not lack of available information about developmental milestones or health issues, but rather one of potentially being overwhelmed. In this context, a visit to a trusted GP could be vital:

*'I like to be reassured … because you know when you have a baby you never know if they’re like normal, healthy and everything is — and you always try to interpret things like oh, is he not smiling, maybe there’s something wrong … you always imagine the worst. So it’s good to have a GP check and reassure you that everything is normal.'* (PA1, mother of 6)

This trust was enhanced with a known GP, in the context of a continuing relationship. Most participants felt that a trained GP, examining their baby, would be able to provide better reassurance than other sources because they are physically checking the baby, rather than providing information about babies in general:

*'I was with the same doctor, erm, which is nice to have that familiarity ... I have trust in her ... I know she has years of experience and she specialises with the babies as well. So that definitely gives that sort of trust and reassurance, that she’s doing a good job and that she knows what she’s doing.'* (PA2, mother of 4)

### Experiences of the appointment

Managing appointments could be frustrating, given the challenges of getting infants (and possibly older children) to a clinic at a set time. A few participants commented on the system for appointments in general, and one participant recounted a particularly frustrating experience involving multiple visits to complete the baby’s registration:

*'... when you have a newborn, how can they expect you to be on the phone at 8.30 sharp and then call back like twenty times ... most of the time she turns round and says, "Oh well, we don’t have anything for today," ... I’m very frustrated with that.'* (PA1, mother of 6)

*'Don't waste my time four times when I've got two children — to try and come in and register my child’s pretty hard work ... they should make it a bit easier.'* (PA8, mother of 2)

Combining the baby check with the mother’s postnatal check or first vaccination to reduce the number of appointments, and systems that simplified registration and proactively sent appointments were positively received:

*'... that was quite efficient. They just sent me the letter with the 6-week check and the first injections, so I thought that was really good actually. So they proactively did that ... I liked the fact that they contacted me before, I didn’t have to make the appointment, they made all the appointments.'* (PA3, first-time mother)

*'I completely forgot about this one, so I got a phone call ... and they registered her through the phone, yeah, so that was really nice, and then they sent the appointment for her so actually, although I forgot about it, they took care of it.*' (PA4, first-time mother)

When they recalled the appointment, most parents thought that the time allocated for the check was sufficient:

*'... it wasn’t rushed. It was quick but it wasn’t rushed at all. Everything that needed to be done, was done, and there was no rush, absolutely none.'* (PA7, mother of 4)

*'... she seemed to take her time over it and she seemed quite thorough, so I think that was all, that all left me feeling kind of reassured.'* (PA3, first-time mother)

However, those that did report feeling rushed felt there was limited time for their concerns or a thorough examination of the baby:

*'... so that’s doctors’ appointments for you ... I feel like they are always rushed, um ... and you're not given the time to sort of take in what’s happened.'* (PA10, mother of 3)

*'... it was very quick and I didn’t really feel like she was examining her at all.'* (PA13, mother of 2)

Participants were generally positive about GPs who explained what they were doing as they went through the physical examination, and commented negatively if this had not been experienced. Given the importance of reassurance about the baby’s normal development, parents did identify this as something they valued being communicated explicitly:

*'... she told me, "You take her clothes off and put her on the scale, I’m going to measure her now. You can just hold her head," you know, that’s the kind of — so I knew what was going on ... if there was a mark I would give everything 10 out of 10.'* (PA7, mother of 4)

*'... maybe if things would have been explained a little more, you know, after each thing they checked, he could have said "Oh that’s great, you know, that’s fine, that’s normal" ... but I felt I had to ask, you know, "Is that good? Like, is she normal?" … so maybe a little more encouragement and explanations.'* (PA11, mother of 4)

The presence of trainees or students could interfere with communication, but it could also flag up the ‘missing’ explanations of the GP:

*'... she only explained, as I said, because of the trainee, so that was the only reason why I overheard what she was doing, otherwise I would have just watched on and I guess it’s important for parents to know what they're doing.'* (PA10, mother of 3)

*'I would have preferred the doctors explained things a bit more to me after each thing they checked ... I didn’t feel so part of it ... they were kind of referring to each other more and conferring ... at some points I did have to ask, if they said, you know, one of their words to each other, I’d be like "What does that mean, is that OK?*"' (PA11, mother of 4)

Four participants reported that their baby’s check had resulted in referral (for ultrasound screening or a specialist opinion). These referrals were not reported as causes of anxiety, but rather as further ‘general checking’ of the infant and reassurance:

*'... we weren’t worried about it ... especially because, you know, like when we were discharged, the midwife did say, "Everything is fine but just for precaution you’re going to have to go to this clinic," so I don’t think we were especially worried*.' (PA14, first-time father)

*'Yeah but she would rather check that* [heart] *just in case.'* (PA4, first-time father)

*'I think they just want to make 100% sure that everything is fine, so I don't feel scared, unhappy or worried ... I think the way the doctor presented it to us was quite, you know, she explained why and what and what it’s based on that makes me feel, you know, informed and easy.'* (PA4, first-time mother)

## Discussion

### Summary

The primary purpose of the 6-week check is screening to identify problems that require further investigation. The findings of this study suggest that it is also often an important event for parents, marking a significant threshold of ‘presenting’ the baby to a trusted source of reassurance on the normal development of the baby, and on the parents’ job to date. Despite being adept at finding information about health and development from other sources, the parents in this study identified a trusted GP, who physically examined their baby, as an important provider of this reassurance. The majority of parents reported positive experiences of the 6-week check.

### Strengths and limitations

A strength of this study is the use of qualitative interviews to explore in-depth the experiences of parents bringing their baby for a recent 6-week check, which is an under-researched area. A range of parents were included, in terms of parity and age, and parents were interviewed within a few weeks of the appointment to maximise recollection. However, a limitation is that all the participants are from general practices in one city that had volunteered to take part; therefore, the experiences parents reported may not be representative of other areas.

### Comparison with existing literature

There is little evidence to date on parents’ views of the 6-week check. Roche *et al* looked at parents’ experiences of child health surveillance overall, and found positive views about the 6-week check, which was seen as comprehensive, compared with the later 8-month check, which was perceived as more bureaucratic.^12^ Talbot *et al*, in a study of GPs’ views of the mother and baby check as an opportunity to instigate and encourage health behaviour change, noted that one barrier to this was that many GPs felt the consultation should be patient-led, and that more research on women’s expectations and experiences was needed.^15^ The findings of the present study corroborate GPs’ orientations towards parent-focused consultation styles for the 6-week baby check. The overwhelming expressed need was for reassurance, and this may not be the best moment for raising health behaviour issues not raised by parents themselves.

Symptoms of depression and anxiety are common in new mothers.^16,17^ The experience of the mother in this study who reported that her GP had missed her needs for support for symptoms of anxiety is consistent with the findings of Johnston *et al^18^* on the importance of GPs listening to patients. It is also consistent with a recent systematic review of the facilitators and barriers to raising emotional concerns in primary care, which identified limited time as a barrier.^19^

### Implications for research and practice

The importance of the 6-week check for parents, and the need for adequate time to provide person-centred care, suggests that continuing to provide long enough appointments for thorough infant screening, reassuring parents, and time for them to raise issues, is essential. Although most practices in this study could and did provide at least a double-length appointment, time pressures may be a barrier for others. Many strategies for managing the workload in primary care focus on greater use of the extended primary care team,^7^ and training other healthcare professionals to undertake the NIPE checks is one area where this has been suggested.^10^ However, it is noted that parents were particularly positive about the GP, a highly trusted professional, doing the check. Any task-shifting should be carefully considered in the light of not just the competence of other professionals to do the NIPE screening, but also to meet the other needs identified. Parents who felt unrushed and that there was a chance to voice concerns were most positive. The 6-week check is an important point of potential intervention for mothers who are experiencing mental health challenges. Even more experienced parents expected and appreciated GPs who provided reassurance about their baby’s normal development, and their own job as parents.

There are implications for practice organisation and for how GPs conduct the consultation. First, practices can proactively register newborn babies when they get notification of the birth. Second, where possible, at least double, combined appointments for mother and baby make the appointment easier to attend and provide opportunities for addressing mother and baby issues holistically. Meeting parents’ expectations to provide parent-focused health care, as well as delivering high quality clinical screening, requires a long consultation.

In terms of clinical practice, GPs can ensure that they share details with the parents and/or carers of what is being examined while doing the 6-week check. To meet parents’ needs, they should provide explicit reassurance about the baby’s development, and parenting ability. The findings of this study reiterate that the baby check can be an important point for identifying concerns, including around the mental health of new parents. GPs can, therefore, ensure the consultations are parent-led, with sufficient time allocated to undertake the full baby screening, and to allow time for parents to ask questions and raise concerns in relation to their own health as well as that of the infant. Finally, while data collection for this study took place with parents relatively recently (the majority of the interviews were in the second half of 2019), it should be acknowledged that the delivery of face-to-face health care in primary care is likely to be subject to change in the future. Consequently, the expectations and experiences of parents are also likely to change. Research evidence, such as that presented here, needs to be revisited on a regular basis.
